# Mercury Levels in Selected Medicines and Dietary Supplements in Poland

**DOI:** 10.1007/s12011-024-04269-3

**Published:** 2024-06-27

**Authors:** Barbara Brodziak-Dopierała, Agnieszka Fischer, Bożena Ahnert

**Affiliations:** Department of Toxicology, Toxicological Analysis and Bioanalysis, Faculty of Pharmaceutical Science, Medical University of Silesia, Ostrogórska 30 Str. 41-200, Sosnowiec, Poland

**Keywords:** Mercury (Hg), Dietary supplements (DS), Medicines, AAS

## Abstract

Current trends are promoting youth, beauty, health, and fitness. Individuals often seek out remedies, such as medicines or dietary supplements (DS), to achieve these goals. However, highly processed foods, chronic stress, and environmental pollution contribute to the development of civilization diseases. The aim of this study was to evaluate the mercury (Hg) content in medicines and DS that are available in Poland. A total of 139 preparations were tested (75 drugs, 64 DS). The medicines contained preparations belonging to antibacterial, antiviral, antifungal; analgesic, antipyretic, and anti-inflammatory; heart and blood vessel disease preventatives; respiratory tract infections treatment; diuretics; aiding digestion; supplements; antidiarrhoeals; anti-allergics; anti-rheumatics; antibiotics; and others. The tested dietary supplements had an effect on the following: improve the condition of skin, hair, and nails; vitamins; minerals; probiotics; weight loss; special for women; and others. The Hg content of the samples was determined using atomic absorption spectrometry (AAS). The Hg content of all the preparations varied widely (0.1–57.4 µg/kg), with a median Hg concentration of 1.2 µg/kg. The median Hg concentration for medicines was 0.8 µg/kg, prescription medicines having higher Hg concentrations (0.9 µg/kg) than over-the-counter (OTC) drugs (0.5 µg/kg). For DS, the Hg content was found to be higher than for drugs, at 2.0 µg/kg. The herbal preparations showed the highest Hg content among the individual DS groups (3.4 µg/kg). The Hg concentrations in the tested drug and DS samples did not exceed acceptable standards. However, if multiple pharmaceutical preparations are taken simultaneously over a long period of time, and there is existing environmental exposure, there is a possibility of Hg concentration accumulation and adverse health effects.

## Introduction

People to improve their physical appearance and overall well-being use dietary supplements (DS). Dietary supplements are products intended for consumption that contain ingredients to supplement the diet. They are a concentrated source of nutrients and other substances that produce a nutritional or physiological effect. They may contain vitamins, minerals, herbs, amino acids, and other dietary substances. DS are preparations, either single or compounded, sold in a variety of forms to facilitate their use, such as tablets, capsules, pills, and lozenges [1, 2]. In European Union countries, DS are subject to the same legal regulation and control as food [1–3]. In the USA, the Food and Drug Administration (FDA) is responsible for controlling the safety of drugs, DS, and food.

A medicine is a substance or mixture of substances used for the prevention and treatment of diseases, as well as for diagnosis and to improve or modify physiological functions of the body. It exhibits pharmacological, metabolic, or immunological effects [4]. Substances found in medicines, such as vitamins and minerals, may also be present in DS. However, in DS, their concentration should not exceed the recommended daily dose [1]. It is important to note that a product cannot have both DS and drug status. Lack of awareness among consumers regarding the differences between drugs and DS can have negative effects on health [5].

Medicines are subject to continuous and rigorous testing throughout their manufacture and distribution to ensure the highest standards of safety and quality are maintained [4, 6, 7]. In contrast, DS do not undergo this type of testing before being re-leased for sale [8]. Medicines and DS are both widely used, but there are important differences in their regulation [9]. The drug market and DS correspond to each other. Additionally, it is worth noting that drug users are more likely to take DS. It is estimated that nearly half of the Polish population periodically or regularly uses DS [10]. The largest portion of the Polish market is held by preparations containing magnesium, immunostimulants, probiotics, body-strengthening preparations, vitamins, and minerals [9].

Herbal supplements are a popular choice for self-medication and bodily function support [11, 12]. However, there is a common misconception that these products are harmless due to their natural ingredients. While they do offer therapeutic benefits, such as antioxidant and anti-inflammatory effects [13–15], it is important to note that adverse effects, including allergic reactions, genotoxicity, carcinogenicity, teratogenicity, organ damage, and even mortality, have been observed [16–20]. The use of products that are inaccurately labelled, contaminated, inhomogeneous, or contain doses of substances that differ from those declared is associated with health risks [5, 21]. Adulterated DS are frequently found on unsupervised online markets and are typically produced outside the EU, mainly in China [5]. Scientific reports indicate that their use in many regions of the world can lead to acute kidney injury due to aristolochic acid, as well as alkaloids, anthraquinones, flavonoids, and glycosides. Some of the Chinese herbs have been described as nephrotoxic, causing acute interstitial nephritis and necrosis [16]. The use of Ayurvedic products is associated with toxicity due to their metal content (As, Hg, and Cr) which is a major concern. This is because there is a lack of regulation regarding the production and purity of these products [17]. Improper dosage or overdose of plant-derived constituents can lead to metabolic activation and subsequent toxicity, which can initiate or exacerbate hepatotoxicity, nephrotoxicity, and pulmonary toxicity. In addition to the natural toxic constituents that are already known, there may be other unidentified substances that are hazardous to the body [18]. Moreover, the simultaneous use of herbal supplements and medications may result in interactions. About one-third of DS users who take medicines reported liver-related adverse effects [19, 20]. Medicinal plants can also contain excessive amounts of heavy metals and pesticides, whether from agricultural use, production, or environmental contamination [22, 23].

Mercury (Hg) is a highly toxic element that has no physiological role in the body. Its affinity for thiol groups, enzymes, amino acids, and sulphur-containing antioxidants renders them inactive, leading to increased oxidative stress and decreased anti-oxidant defenses. Hg also causes mitochondrial dysfunction by lowering ATP levels and decreasing glutathione levels and increases lipid peroxidation [24–27]. Hg may have carcinogenic properties. In experimental models, exposure to low doses of Hg has been shown to induce a proliferative response in normal and tumour cells by interfering with oestrogen receptor, ERK1/2 (protein kinase), JNK (N-terminal kinase), and NADPH-oxidase [28]. High hair Hg levels have been observed to be correlated with inflammatory myofibroblastic tumour of the carotid artery and carotid atherosclerosis [29]. Furthermore, an association was found between high fibrinogen levels, low HDL cholesterol levels, and high blood pressure, which may increase the risk of stroke and cerebrovascular accident (CVA) events [29–31].

The study aimed to evaluate the Hg content in medicines and DS marketed and sold in Poland. In several countries, including Poland, there is a growing trend of following a ‘healthy’ diet, which involves consuming nutrient-rich products, including readily available DS preparations. This pursuit of an improved quality of life has led to a significant increase in the use of DS. The use of DS and medicines, despite their health benefits, can result in exposure to harmful substances. These indications prompted a study to be undertaken. Under Polish legislation, DS can be purchased without any restrictions or controls [1]. Prescription drugs can only be purchased at designated points of sale, such as pharmacies and pharmacy outlets. Some medicines, known as over-the-counter (OTC) medicines, do not require a doctor’s prescription and can be purchased in places other than designated medicine outlets, such as drugstores, grocery shops, and petrol stations. Taking medicines may vary depending on medical indications or personal factors such as the patient’s age or health condition. Dosage instructions should be followed carefully. The use of DS should be temporary or long-term as directed. The factors mentioned can influence the dosage of the medication, including the amount of Hg taken with it.

The study aimed to determine whether the amount of Hg found in the tested samples exceeded acceptable standards. Compare the levels of Hg, in DS and various types of drugs available for sale, both over-the-counter and by prescription. The study identified the preparations with the lowest and highest levels of Hg.

## Materials and Methods

### Samples

The study focused on popularly used medicines and DS in Poland. The study analysed 139 preparations, including 75 drugs and 64 DS, which were obtained from various points of sale in Poland between 2022 and 2023. The medicines were purchased exclusively from pharmacies, while the DS were purchased from pharmacies, drug-stores, hypermarkets, and online shops.

The surveyed medicines included 48 prescription and 27 OTC.

The DS were categorised based on their composition (herbal, vitamin-mineral, or other) and indication for use (minerals, vitamins, probiotics, improving the condition of skin, hair, and nails, for women, weight loss, or other) (Table 1).

**Table 1:** Characteristics of tested dietary supplements

Name	Active substances	Grp
Vita-min plus	Minerals: Ca, Mg, K, Zn, Mn, Fe, Cu, Se, Cr, vitamins: C, E, A, B_6_, B_1_, B_2_, B_12_, niacin, biotin, pantothenic acid, pteroylmonoglutamic acid, dong Quai ginseng extract (*Angelica sinensis* L.), soy isoflavones extract, black pepper extract (*Piper nigrum* L.)	1
Dervit	Chicory inulin, vitamin-mineral complex (L-ascorbic acid, retinyl acetate, DL-alpha-tocopheryl acetate, thiamine hydrochloride, riboflavin, pyridoxine hydrochloride, D-biotin, nicotinic acid amide, zinc oxide, iron (II) gluconate), field horsetail (*Equisetum arvense* L.) stems and leaves extract, corn starch-carrier, nettle (*Urtica dioica*) leaves extract	
Bratek plus	Extract from the herb of violet tricolor (*Viola tricolor* L.), zinc	
Belissa	Horsetail herb extract, vitamins: D, B_1_, B_2_, B_12_, minerals: Fe, Zn, Ca, L-cysteine, lactose, DL-alpha-tocopheryl acetate, retinyl acetate, gelatin, vegetable oil, pyridoxine hydrochloride	
Skrzyp polny forte	Zinc, extract of field horsetail herb (*Equisetum arvense* L.), extract of common nettle herb (*Urtica Dioica* L.), vitamins: A, B_2_, B_1_, B_6_, D_3_, folic acid, biotin	
Skrzyp strong	Horsetail herb extract (*Equisetum arvense* L.), vitamins: C, B_1_; zinc; nettle herb extract (*Urtica dioica* L.); silicon dioxide; biotin	
Skrzyp optimal	Horsetail extract, zinc gluconate, L-ascorbic acid, nettle leaf extract, magnesium stearate, thiamine hydrochloride, D-biotin	
Vitalss plus	Calcium D-pantothenate, vitamins: B_6_, B_2_, B_1_, B_12_, folic acid	
Wyciąg ze skrzypu polnego i pokrzywy	Field horsetail, nettle, vitamins: C, A, E, B_1_, B_6_	
Merz special	Minerals: Fe, Ca, Zn, vitamins: C, B_6_, B_1_, B_2_, A, B_1_, E, folic acid, D-biotin	
Revalid	DL-methionine, wheat germ extract, millet extract, L-cystine, brewer’s yeast, para-aminobenzoic acid, minerals: Ca, Fe, Zn, Cu, thiamine hydrochloride, biotin, vitamin D	
Beauty skrzyp	Horsetail herb extract (*Equisetum arvense* L.), nettle herb extract (*Urtica dioica* L.)	
Maxi krzem	Field horsetail herb extract, bovine gelatin, nettle herb extract, bamboo shoot extract, nicotinic acid amide, vitamins: C, B_1_, B_2_, B_12_, D, minerals: Fe, Zn, Mn, K, Se, L-methionine, pantothenic acid	
Zajavit	Vitamins: C, E, B_2_; iron oxides and hydroxides, magnesium salts of fatty acid	
Skrzyp complex	Horsetail herb extract (*Equisetum arvense* L.), nettle herb extract (*Urtica dioica* L.); biotin vitamins: C, A, B_1_; Zn	
Vita-min plus senior	Vitamins: A, D, E, C, B_2_, B_6_, biotin, minerals: Si, Mn, Zn, Cu, Se	2
Sundovit D3 + K2	Vitamins: D, K	
Body vitality complex	Vitamins: A, D, E, K, C, B_1_, B_2_, B_12_, minerals: Fe, Zn, Cu, K, Se, Cr, ginseng root extract (*Panax ginseng*)	
Witagin	Ginseng root, vitamins: E, B_1_, B_2_, B_6_, B_12_, PP, C, A, D_3_ folic acid, biotin, minerals: Fe, Zn, Cu, Mn, Cr, Mg, Se	
Calcenato	Calcium carbonate, magnesium oxide, zinc	
Ona-a-day	Vitamins: A, E, B_1_, B_6_, B_12_, B_2_, C thiamine, biotin, folic acid,minerals: Ca, P, Fe, J, Mg	
Omega med. odporność	Honey, tocopherol, esters of fatty acids, vitamin C	
Vitamin C allnutrition	Vitamin C, bioflavonoids	
Witamina C1000	Vitamin C	
Ceviforte C1000	Vitamin C	
Vita-miner	Vitamins: E, B_1_, B_6_, B_12_, B_2_, C, biotin, niacin, pantothenic acid, folic acid, Fe, Zn, rutin	
Molekin D3	Vitamin D_3_	
Multiwitamina complex	Vitamins C, E, B_6_, K_2_, D_3_, B_1_, B_6_, B_12_, pantothenic acid, folic acid	
Rutinacea complete	Vitamin C, rutoside, zinc, selenium, citrus bioflavonoids	
Rutinacol	Vitamin C, rutin, Zn, garlic extract	
Revitaben complex + żeń-szeń	Vitamins: C, E, B_6_, B_12_, D, B_2_; niacin, thiamin, pantothenic acid, folic acid	
Multivital complex	Vitamins: E, B_2_; niacin, biotin	
Falvit estro	Vitamins: C, E, niacin, hops cone extract	
Bilomag plus	Vitamin B_6_, ginkgo biloba extract, lecithin, Mg	
Vitalsss complex 50 +	Vitamins: C, E, B_2_, B_6_	
Vita strong	Vitamins: E, C, A, E, B_1_, B_2_, B_6_, niacin, biotin	
Multivitamin	Vitamins: A, D, E, C, E, B_1_, B_2_, B_6_, B_12_, niacin, biotin, folic acid	
Artresan optima	Minerals: Zn, Mn, vitamin C, glucosamine sulphate, niacin, type II Collagen, chondroitin sulphate, ginger root extract, turmeric rhizome extract, rosehip extract	3
Flexi protect	Vitamin C, collagen, chondroitin, hyaluronic acid, ginger rhizome extract	
Biotynox max	Biotin, Zn, Se	
Magene vit.B_6_	Magnesium, vitamin B_6_	
Neomag young	Magnesium, vitamin B_6_, lecithin	
Kerabione	Minerals: Zn, Cu, Se, L-cysteine, L-lysine, L-methionine, vitamins: C, A, E, B_2_, B_3_, bamboo sprout extract, hyaluronic acid, biotin	
Zincuprin	Zn, Cu	
Biotyk	Inulin, lactic acid bacteria (*Lactobacillus rhamnosus* GG)	4
Trilac lady	*Lactobacillus plantarum*, *Lactobacillus rhamnosus*, *Lactobacillus paracasei*	
Probiotyk	lactic acid bacteria (*Lactobacillus rhamnosus* GG)	
Iladian	L-ascorbic acid, inulin, vitamin E, hyaluronic acid	
Diarof	*Saccharomyces boulardii*, nucleotides, inulin	
Vitalsss plus	*Garcinia cambogia* (Tamarind Malabar HCA)	5
Chrom aktiv	Green tea extract	
Młody jęczmień Forte Slim	Young barley extract (*Hordeum vulgare*) bitter orange fruit extract (*Citrus aurantium*), biotin, chromium	
Młody jęczmień- Green Magma	Young barley extract, bitter orange fruit extract	
Verdin complex	Rosemary leaves and rosemary leaf extract, artichoke leaves and artichoke leaf extract, extract of long oyster rhizomes peppermint oil	
Hepatrim	Artichoke, chlorella, black radish	
Climea forte	Soybean seed extract, hops cone extract, flax seed, calcium, vitamins: D3, B6, E, folic acid	6
Ogestan	Green tea extract, folic acid, vitamin D, Cr	
Novovit duo	Folic acid, vitamins: B_6_, B_1_, B_2_	
Climeston	Red clover leaf and flower extract (*Trifolium protense* L.), vitamins: K, D_3_, calcium	
Soyfem forte	Glycine max semen extract	
Złocień maruna	Marunas thistle	7
Aviomarin natural	Ginger rhizome extract	
Diabetostrong	White mulberry (*Morus alba* L.) leaf extract, *Gymnema sytvestre* leaf extract, Ceylon cinnamon (*Cinnamomum verum*), bark extract	
Nervoton	Melissa leaf extract, chamomile flower extract, *Rhodiola rosea* root extract, Mg, vitamin B_6_	

*Grp.*, group of DS: 1—improve the condition of skin, hair, and nails, 2—vitamins, 3—minerals, 4—probiotics t, 5—slimming, 6—special for women, and 7—other

In the case of medicines, they are divided based on their action. These actions include antibacterial, antiviral, anti-fungal; analgesics, antipyretics, anti-inflammatories; heart and blood vessel disease preventatives; respiratory tract infection treatments; diuretics; aiding digestion; supplements; antidiarrhoeals; anti-allergics; anti-rheumatics; antibiotics; and others (Table 2).

**Table 2:** Characteristics of tested drugs

Name	Active substance	Recept	Grp	Indication
Groprinosin forte	*Inosine pranobex*	No	1	Supportively for recurrent upper respiratory tract infections; in infections with viruses
Furaginum	*Furazidinum*	No		Treatment of lower urinary tract infections caused by *Escherichia coli*
Xifaxan	*Rifaximinum*	Yes		Intestinal infections, with rifaximin-sensitive bacteria
Femannose N	*D-Mannose*	No		Treatment of cystitis
Dafurag max	*Furazidinum*	No		Infection of the lower urinary tract
Metafen	*Ibuprofenum* + *Paracetamolum*	No	2	Pains of various origins, fever
Etopiryna	*Acidum acetylsalicylicum* + *Coffeinum*	No		Pain of weak or moderate intensity, fever
Polopiryna S	*Acidum acetylsalicylicum*	No		Pain of various origins of mild to moderate intensity, fever
Efferalgan codeine	*Paracetamolum* + *Codeini phosphas*	Yes		Pain of moderate to severe intensity
Paracetamol	*Paracetamolum*	No		Pain of various origins of mild to moderate intensity, fever
Aspiryn	*Acidum acetylsalicylicum*	No		Pain of low to moderate intensity, fever
Ibuprom zatoki	*Ibuprofenum* + *Pseudoephedrini hydrochloridum*	No		Alleviation of symptoms occurring in cold and flu
Ketonal forte	*Ketoprofenum*	Yes		Treatment of inflammatory, degenerative and metabolic rheumatic diseases and relief of pain syndromes
Ibuprom	*Ibuprofenum*	No		Used to treat inflammation, which is one of the causes of pain. The drug reduces fever
Acard	*Acidum acetylsalicylicum*	No	3	Long-term, prophylactic use in diseases that threaten to formation of clots and congestion in the blood vessels
Proficar	*Acidum acetylsalicylicum*	No		Inhibition of platelet aggregation
Cardioteva	*Acidum acetylsalicylicum*	No		Inhibition of platelet aggregation
Gardimax medica	*Chlorhexidini digluconatis solutio* + *Lidocaini hydrochloridum*	No	4	Inflammatory conditions of the mouth and throat
Thiocodin	*Codeini phosphas hemihydricus* + *Sulfogaiacolum*	No		Treatment of dry, persistent cough without expectoration of secretions
Flavamed	*Ambroxol hydrochlorid*	No		Mucolytic treatment in acute and chronic diseases of the bronchi and lungs
Erdomed	*Erdosteinum*	Yes		Secretolytic treatment in acute and chronic diseases of the upper respiratory tract of the respiratory tract, bronchi and lungs
Spironol	*Spironolactonum*	Yes	5	Kidney disorder causing excessive fluid in the body
Finospir	*Spironolactonum*	Yes		Reduces excess fluid in the body by increasing urine excretion
Diuver	*Torasemidum*	Yes		Edema due to congestive heart failure, pulmonary edema, edema of hepatic origin hepatic and renal
Furosemid	*Furosemidum*	Yes		Treatment of edema associated with congestive circulatory failure, with cirrhosis of the liver and kidney disease
Verospiron	*Spironolactonum*	Yes		Diuretic drug with diuretic effect
Toradiur	*Torasemidum*	Yes		Treatment and prevents recurrence: fluid retention in tissues, fluid retention in body cavities
Torsemed	*Torasemidum*	Yes		Treatment of edema, which is caused by excessive water in the body
Furosemid	*Furosemidum*	Yes		Treatment of edema associated with congestive circulatory failure, with cirrhosis of the liver and kidney disease
Verospiron	*Spironolactonum*	Yes		Diuretic drug with diuretic effect
Trifas Cor	*Torasemidum*	Yes		Swelling and transudates
Ircolon	*Trimebutini maleas*	Yes	6	Motility disorders and intestinal complaints associated with functional disorders of the gastrointestinal tract
Debretin	*Maleinian trimebutyny*	Yes		Gastrointestinal motility disorders
Kreon	*Pancreatinum*	Yes		Treatment of digestive disorders in the course of exocrine pancreatic insufficiency
Enterol	*Saccharomyces boulardii CNCM I-745*	No		Treatment of acute infectious diarrhoea and prevention of post-antibiotic diarrhoea
Rutinoscorbin	*Rutosidum trihydricum* + *Acidum ascorbicum*	Yes	7	States of deficiency and increased demand for vitamin C
Calperos	*Calcii carbonas*	No		States of increased demand for calcium:
Terdyferon	*Ferrosi sulfas*	Yes		Treatment of iron deficiency anaemia and in the prevention of iron deficiency
Aspargin	*Magnesii hydroaspartas* + *Kalii hydroaspartas*	No		Supplementation of magnesium and potassium deficiencies
Carbo medicinalis	*Carbo activatus*	No	8	Treatment of diarrhoea, indigestion and flatulence; in poisoning by drugs and other chemical compounds
Carbo medicinalis	*Carbo activatus*	No		Treatment of diarrhoea, indigestion and flatulence; in poisoning by drugs and other chemical compounds
Loperamid wzf	*Loperamidi hydrochloridum*	Yes		Symptomatic treatment of acute and chronic diarrhoea
Alax	*Aloe capensis* + *Frangulae corticis extractum siccum*	No		For short-term use in occasionally occurring constipation
Clatra	*Bilastinum*	Yes	9	Relieve symptoms of hay fever and other forms of allergic rhinitis
Clemastinum WZF	*Clemastinum*	Yes		Alleviating allergic symptoms, especially of the skin and nasal mucosa
Telfexo	*Fexofenadini hydrochloridum*	Yes		Alleviation of hay fever symptoms
Allertec	*Cetirizini dihydrochloridum*	No		Relief of nasal and ocular symptoms associated with seasonal and chronic allergic rhinosinusitis
Delortan allergy	*Desloratadyna*	Yes		Relief of nasal and ocular symptoms associated with seasonal and chronic allergic rhinosinusitis
Diclo duo	*Diclofenacum natricum*	Yes	10	Inflammatory forms of rheumatic diseases
Chronada	*Chondroitin sodium sulphate* + *Glucosamine hydrochloride*	Yes		Treatment of osteoarthritis of the knee joint
Metindol retard	*Indometacinum*	Yes		treatment of acute rheumatoid arthritis
Trosicam	*Meloxicamum*	Yes		Short-term treatment of symptoms of exacerbation of osteoarthritis and long-term treatment of symptoms of rheumatoid arthritis
Anapran ec	*Naproxenum*	Yes		In the symptomatic treatment of rheumatoid arthritis
Amoksiklav	*Amoxicillinum* + *Acidum clavulanicum*	Yes	11	Sinus infections, urinary tract infections, skin infections, dental infections
Zinacef	*Cefuroximum*	Yes		Treatment of infections of the lungs or chest, urinary tract, skin and soft tissues, abdomen
Klabax	*Clarithromycinum*	Yes		Treatment of infections caused by microorganisms sensitive to clarithromycin
Clindamycin—MIP	*Clindamycinum*	Yes		Bacterial infections caused by clindamycin-susceptible strains
Ospamox	*Amoxicillinum*	Yes		Treatment of infections of various parts of the body caused by bacteria sensitive to amoxicillin
Ceroxim	*Cefuroximum*	Yes		Treatment of infections of the throat, sinuses, middle ear, lungs or chest, urinary tract, skin and soft tissues
Zinnat	*Cefuroximum*	Yes		Treatment of infections of the throat, sinuses, middle ear, lungs or chest
Tacefur	*Cefuroximum*	Yes		Treatment of infections of the throat, sinuses, middle ear, lungs or chest
Duomox	*Amoxicillinum*	Yes		Treatment of infections caused by bacteria sensitive to amoxicillin
Taromentin	*Amoxicillinum* + *Acidum clavulanicum*	Yes		Treatment of middle ear and sinus infections, respiratory tract, urinary, skin, soft tissue, bone and joint
Cipronex	*Ciprofloxacinum*	Yes		Infections caused by Gram-negative bacteria
Amoyess	*Amoxicillinum*	Yes		Treatment of infections caused by bacteria sensitive to amoxicillin
Ramoclav	*Amoxicillinum* + *Acidum clavulanicum*	Yes		Infection of the middle ear and paranasal sinuses, respiratory tract, urinary tract, skin, soft tissues, bones and joints
Augmentin	*Amoxicillinum* + *Acidum clavulanicum*	Yes		Treatment of middle ear and sinus infections, respiratory tract, urinary, skin, soft tissue, bone and joint
Mydocalm	*Tolperyzon*	Yes	12	Treatment of pathologically increased muscle tone Skeletal muscles after stroke in adult patients
Tramal Retard	*Tramadoli hydrochloridum*	Yes		Indicated for the treatment of moderate to severe pain
Zofenil	*Zofenoprilum calcicum*	Yes		High blood pressure (hypertension), heart attack
Altacet	*Aluminii acetas tartras*	No		Topically on the skin to reduce swelling after tissue and joint contusions
No-spa	*Drotaverini hydrochloridum*	No		Diastolic, reducing pain in the organs of the abdominal cavity abdominal and pelvic organs
Nolpaza	*Pantoprazolum*	Yes		Treatment of gastrointestinal diseases associated with excessive secretion of hydrochloric acid

*Grp.*, group of drugs: 1—antibacterial, antiviral, antifungal, 2—analgesic, antipyretic, and anti-inflammatory, 3—heart and blood vessel disease preventatives, 4—respiratory tract infections treatment, 5—diuretics, 6—supports digestion, 7—supplements, 8—antidiarrhoeals, 9—anti-allergics, 10—anti-rheumatics, 11—antibiotics, and 12—other

The preparations studied varied greatly in terms of usage. They included medicines taken regularly in fixed doses for a specific short period of time (e.g. antibiotics), medicines sometimes used for many years (e.g. those protective against heart and blood vessel disease, anti-rheumatic drugs), as well as drugs used on an ad hoc basis (e.g. antidiarrhoeals, antipyretics, and analgesics).

### Sample Preparation

Before testing, the preparations were stored at room temperature (15–25 °C) out of direct sunlight, as instructed by the manufacturer. A random sample of approximately 40 mg was taken from each bulk preparation package for testing. The preparations for testing were mainly in tablet form (*N* = 99), capsules (*N* = 26), and other forms, such as powder or granules (*N* = 14). The material to be tested was ground to obtain a homogeneous form using an analytical mill (Analytical mill A 11 basic, IKA, Warszawa, Poland). Three independent samples of approximately 100 mg (analytical balance RADWAG, Radom, Poland) were taken from one DS/medicine and analysed.

### Determination of Hg

The total Hg content of the test samples was determined using atomic absorption spectrometry (AAS) by AMA254 analyser (Altec, Praha, Czechy). The detection apparatus used is capable of detecting all forms of Hg present in the test sample. The following measuring conditions were used: wavelength 253.65 nm, carrier gas—oxygen (O_2_ purity ≥ 99.5%), and inlet pressure 200–250 kPa. The measurement technique allows the determination of the total Hg content regardless of its form in the sample. The time of each analytical step was [second]: drying 200, decomposition 250, and measurement 90. The lower detection limit (LOD) is 0.11 µg/kg. Prior to each measurement, the apparatus was cleaned with air and distilled water in accordance with the analytical procedure [32, 33]. The original factory calibration was valid for instrument calibration. The certified reference material (CRM) were used to ensure quality control of the method. Mixed Polish Herbs INCT-MPH-2 was used [34], result from six analysis: 0.018 ± 0.002 mg/kg, recovery 92.22%.

### Statistical Analysis

The results of the determinations underwent statistical analysis using Microsoft Excel and Statistica ver. 13.3 pl (Statsoft, Cracow, Poland). The final concentration of Hg in each test sample was calculated as the arithmetic mean of its three measurement results. The distribution of variables was evaluated using the Shapiro–Wilk test and quantile–quantile plot. The statistical variability was compared using the nonparametric Mann–Whitney *U* test (for two samples) and Kruskal–Wallis test (for a greater number of samples) due to the non-normal distribution of samples. A *p*-value of less than 0.05 was considered statistically significant [35]. The interval data were ex-pressed as a median (Me) and lower (Q1) and upper quartiles (Q3). Additionally, the arithmetic mean (AM), standard deviation (SD), minimum (Min) and maximum (Max) level, and coefficient of variation (CV) were calculated.

### Quality Control

In order to ensure the reliability of the results obtained, quality control procedures were followed during the analysis. Blanks, duplicates and certified reference materials (CMR) were used during the analysis.

Quality control report AMA254 contains calibration curves and results of tests:

- test DL (test of the detection limit),

- test RSD (test of the standard deviation of the instrument),

- recovery test.

## Results

In all drug and DS samples tested (*N* = 139), the mean Hg (AM) concentration was 3.3 µg/kg, with a range of variation (Min–Max) from 0.1 to 57.4 µg/kg. The median Hg content (Me) was 1.2 µg/kg, with a quartile range (Q1–Q3) of 0.5 to 3.8 µg/kg. The Hg content of the study preparations varied and coefficient of variation (CV) was greater than 100% for both medicines and DS. There were no statistically significant differences between Hg concentrations in all medicine and DS samples tested (Table 3). The discussion of the study results was based on the median value (Me). The median concentration of Hg was higher in DS (2.0 µg/kg) than in medicines (0.8 µg/kg) as shown in Table 3.

**Table 3:** Statistical analysis of Hg content in medicines and DS (µg/kg)

	Kind	*N*	AM ± SD	Me	Quartiles		CV [%]
					Q1	Q3	
All		139	3.3 ± 6.9	1.2	0.5	3.8	207
Medicines	All	75	3.9 ± 3.0	0.8*	0.4	2.8	232
	Over-the-counter (OTC)	29	2.5 ± 6.2	0.5	0.4	1.7	246
	Prescription	46	4.7 ± 10.3	0.9	0.5	5.1	219
DS	All	64	2.7 ± 9.0	2.0*	0.8	3.9	110
	Herbal	27	3.9 ± 3.8	3.4	1.4	4.9	97
	Vitamin-mineral	30	1.8 ± 1.7	1.2	0.4	3.3	90
	Other	7	1.1 ± 1.1	0.8	0.7	0.9	93

*N* number of samples, *AM* arithmetic mean, *SD* standard deviation, *Min* minimum, *Max* maximum, *Me* median, *Q1* first quartile, *Q3* third quartile, *CV* coefficient of variation

^*^No statistically significant differences between Hg concentrations in all medicines and DS, *p* = 0.064 (Mann–Whitney *U* test)

**Fig. 1:**
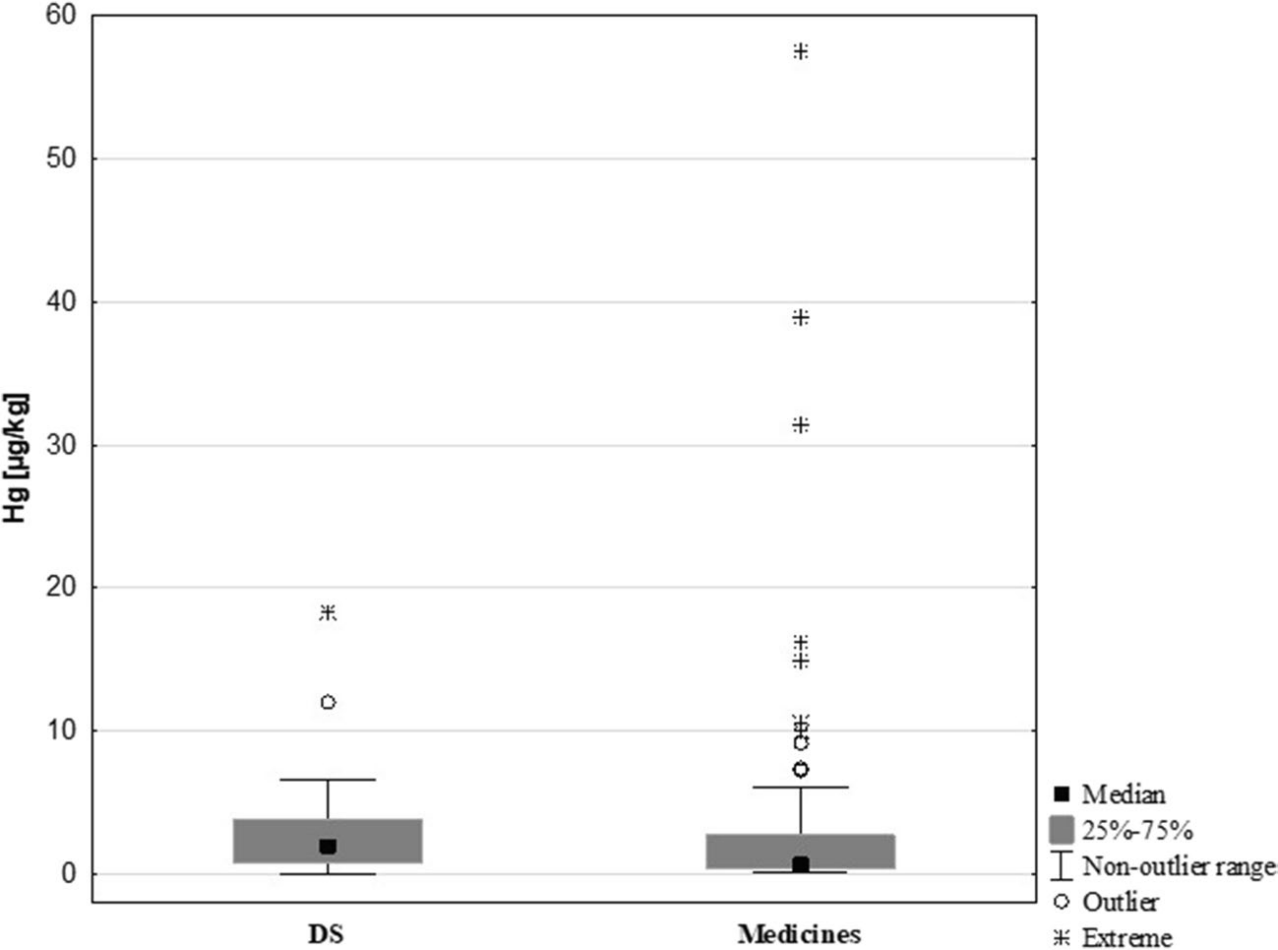
Comparison of the Hg content in medicines and DS

**Fig. 2:**
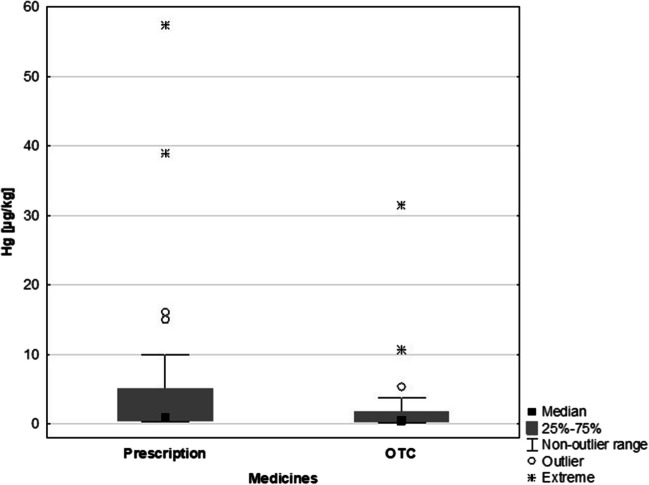
The Hg content in prescription and over-the-counter (OTC) medicines

Analysis of Hg concentrations in the study samples identified several outliers. Figure 1 shows a comparison of the Hg contents in drugs and DS. The Hg contents in DS were more tightly clustered around the mean value than in medicines. Among the medicine samples, there were more Hg concentrations that deviated from the mean value and extreme concentrations, including contents > 30 µgHg/kg. The Hg content of prescription and OTC medicines was subsequently analysed, revealing that Hg concentrations were higher in prescription drugs (0.9 µg/kg) than in OTC (0.5 µg/kg). The groups did not show statistically significant differences, as indicated by the *p*-value of *p* = 0.05 (Fig. 2).

The obtained value, which is close to the significance level (*p* < 0.05), illustrates a statistical trend within the groups. When comparing all the preparations studied and their commercial availability (prescription or OTC), the Hg concentrations developed in the following order: DS (2.0 µg/kg) > prescription drug (0.9 µg/kg) > OTC (0.5 µg/kg) (Table 3).

**Fig. 3:**
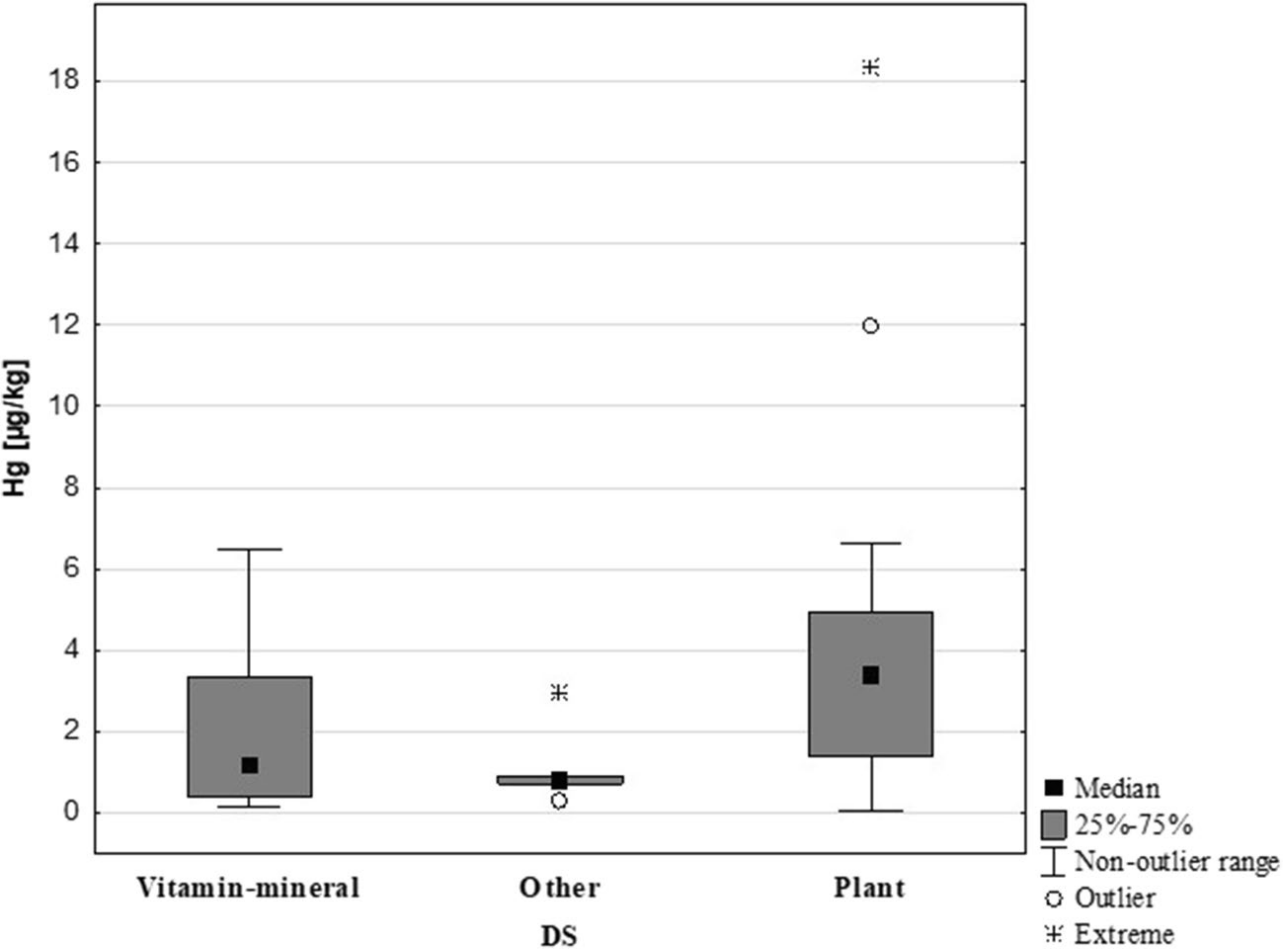
Comparison of Hg content in DS by their composition

The analysis examined the composition of DS, which included herbs, vitamin-mineral, and other preparations (Fig. 3). Statistical analysis revealed significant differences in Hg concentration among the different DS types. The highest Hg content was found in herbal preparations (3.4 µg/kg), which was several times higher than the other DS types. The highest Hg content was found in herbal preparations (3.4 µg/kg); this value was several times higher than other types of DS. Vitamin-mineral preparations had an Hg concentration of 1.2 µgHg/kg, while the ‘other’ preparations had 0.8 µgHg/kg (Table 3). The herbal DS group had an extreme Hg concentration of 18.4 µg/kg (Fig. 3).

The medicine and DS samples were classified according to their indication of use, as described in the literature [36]. The ‘other’ group for DS and medicines includes preparations that do not fit into the distinguished groups. Table 4 presents a list of the distinguished groups with their designated Hg content. The distinguished DS include minerals, vitamins, and supplements that improve the condition of skin, hair, and nails. Additionally, probiotics, slimming preparations, and preparations with a special purpose for women were identified.

**Table 4:** Content of Hg in different in different groups of medicines and DS (µg/kg)

Preparation	Kind	*N*	AM ± SD	Me	Q1	Q3
Medicines	Antibacterial, antiviral, antifungal	5	0.9 ± 1.1	0.5	0.3	0.7
	Analgesic, antipyretic, and anti-inflammatory	9	2.9 ± 4.0	1.1	0.5	1.8
	Heart and blood vessel disease preventatives	3	0.3 ± 0.1	0.2	0.2	0.5
	Respiratory tract infections treatment	4	2.1 ± 3.5	0.4	0.3	3.9
	Diuretics	10	1.1 ± 2.2	0.4	0.4	0.5
	Aiding digestion	4	0.7 ± 0.7	0.4	0.4	1.1
	Supplements	4	2.1 ± 2.7	0.9	0.4	3.8
	Antidiarrhoeals	4	2.2 ± 2.3	1.4	0.7	3.6
	Anti-allergics	5	1.8 ± 2.0	0.9	0.8	1.3
	Anti-rheumatics	5	1.6 ± 2.0	0.7	0.5	1.2
	Antibiotics	14	10.3 ± 17.2	2.2	0.8	15.0
	Other	6	8.6 ± 11.7	4.4	0.9	9.9
DS	Improve the condition of skin, hair, and nails	15	3.4 ± 2.0	3.9	1.3	4.9
	Vitamins	22	1.6 ± 1.4	1.3	0.4	2.7
	Minerals	7	2.2 ± 2.4	0.9	0.4	4.5
	Probiotics	5	1.1 ± 1.1	0.8	0.7	0.9
	Weight loss	6	5.7 ± 6.4	3.9	2.5	5.3
	Special for women	5	2.4 ± 1.5	2.4	0.9	3.9
	Other	4	4.2 ± 5.2	2.0	1.1	7.3

*N* number of samples, *AM* arithmetic mean, *SD* standard deviation, *Me* median, *Q1* first quartile, *Q3* third quartile

**Fig. 4:**
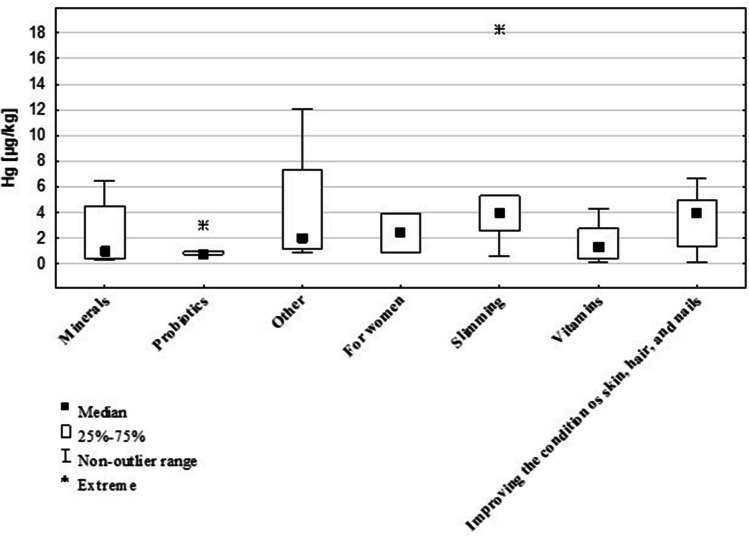
The Hg content in different groups of DS

The study found no statistically significant differences in Hg content between the different DS groups). The highest concentrations of Hg were recorded in DS marketed for weight loss (3.9 μg/kg) and improving the condition of skin, hair, and nails (3.9 μg/kg), while the lowest concentrations were found in probiotics (0.8 μg/kg) and minerals (0.9 μg/kg) (Fig. 4).

**Fig. 5:**
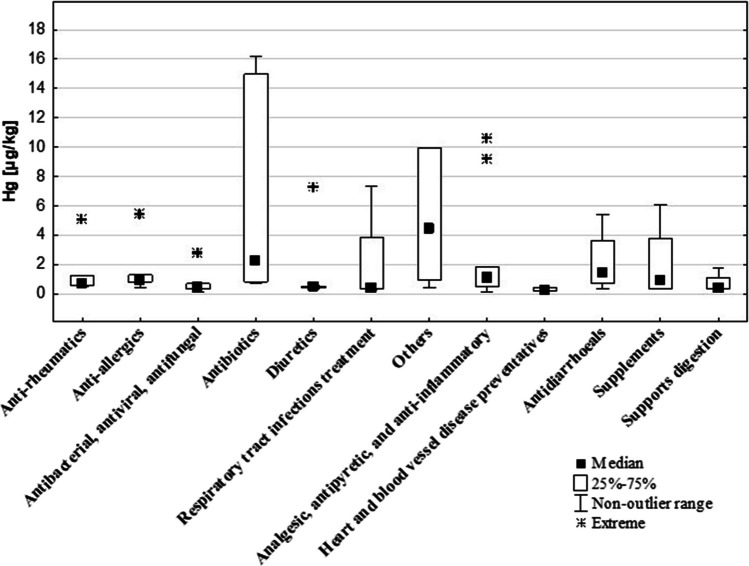
The Hg content in different groups of medicines

Table 4 presents the analysis of Hg content in different medicine groups, while Fig. 5 illustrates the distribution of Hg content among these groups. The group ‘other’ had the highest Hg content (4.4 µg/kg). This group included the medications: diastolic, reduce hydrochloric acid secretion, topical astringents, and reduce increased skeletal muscle tension. Lower Hg concentrations were found in antibiotics (2.2 µg/kg), antidiarrhoeal drugs (1.4 µg/kg), and medicines with analgesic, antipyretic, and anti-inflammatory effects (1.1 µg/kg). Antibiotics had the largest range of Hg content among the distinguished medicine groups. The medicines categorised into the groups of cardiovascular disease prevention (0.2 µg/kg), respiratory tract infections (0.4 µg/kg), and diuretics (0.4 µg/kg) had the lowest Hg concentrations. The differences in Hg concentrations among the different drug groups were statistically significant (*p* < 0.01). The group of drugs recommended for supplementation included macro- and microelements and vitamins, used e.g. in the treatment of osteoporosis and colds. The Hg content in the supplementation medicines was 0.9 µg/kg, which was lower than the average Hg content in all DS tested (Me = 2.0 µg/kg) and comparable to the Hg concentration in vitamin-mineral DS (Me = 1.2 µg/kg) (Table 3).

**Fig. 6:**
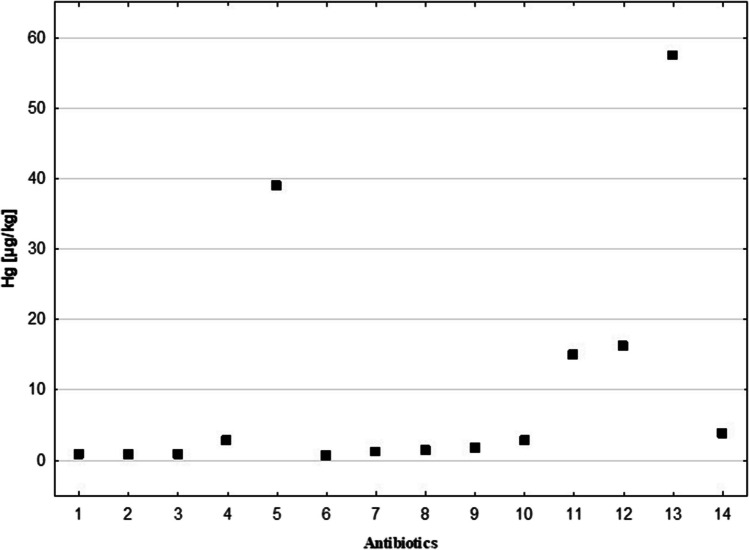
The Hg content in the studied antibiotics, *N* = 14

The concentration of Hg in antibiotics was found to be 2.2 µg/kg, which was the highest among the medicine groups studied, and greater than the Hg content in DS (2.0 µg/kg). Antibiotics also had the highest range of Hg content among the drug groups, with an upper percentile (Q3) Hg content of 15.0 µg/kg (Fig. 5). A detailed analysis of the Hg content of the 14 antibiotics studied is presented in Fig. 6. It was discovered that half of the antibiotics tested had a higher Hg content than the average Hg content for all drugs studied (i.e. 2.0 µg/kg). In three of the preparations, the Hg content ranged from 2.8 to 3.8 µg/kg, and in two, it was about 15 µg/kg, (exactly 15.0 and 16.2 µg/kg). Extreme Hg content was found in two of the antibiotics tested (38.9 and 57.4 µg/kg), both of which contained amoxicillin, a broad-spectrum bactericidal agent.

## Discussion

The popularity of DS is increasing, and market forecasts suggest this trend will continue. It is important for consumers to differentiate between medicinal products and DS. The production and marketing of these products raise concerns about safety, composition, quality, and their effects [5]. Additional confusion arises from over-the-counter (OTC) medicinal products, which, like DS, are available both in and out of pharmacies. In addition to the lack of quality control, DS are not monitored for interactions with other products or their side effects [9]. Furthermore, DS are often consumed over a long period, and their prevalence in advertising implies benefits that may not be supported by evidence. Testing of raw materials and finished products, such as metals and pesticide residues, is necessary to eliminate low-quality DS from the market and ensure a higher level of safety. Currently, there is ongoing discussion regarding greater control of DS. It is important to raise consumer awareness [8]. The Commission Regulation of 2 July 2008, which amends Regulation (EC) No 1881/2006 setting maximum levels for certain contaminants in foodstuffs, sets the maximum level for Hg in DS at 0.1 mg/kg (100 µg/kg) [36]. None of the medicine and DS preparations tested exceeded this value.

Kowalski and Frankowski [37] conducted a study on Hg concentrations in medicines and DS available on the Polish market. The study found that the range of Hg concentrations was very wide, from 0.9 to 476 µg/kg, which is larger than the range found in our study (0.1–57.4 µg/kg). Prescription medicines had the highest Hg content (7.4 µg/kg), followed by OTC medicines (6.0 µg/kg), and DS had the lowest (5.9 µg/kg). In our study, DS had the highest Hg concentration (Me = 2.0 µg/kg). In the study by Kowalski and Frankowski [37], it was found that prescription medicines contained more Hg (0.9 µg/kg) than OTC (0.5 µg/kg), and this difference was statistically significant. The highest median value (16.3 µg/kg) for Hg concentrations in individual drug groups was found in the group of tranquillizers, sleeping pills, antidepressants, anxiolytics, then for antihypertensives, anticoagulants; for ischaemic heart disease, diuretics and inhibitors of gastric acid secretion, also in the same study by Kowalski and Frankowski [37]. In our study, the group of drugs classified as ‘other’ (including decongestants, antacids, topical astringents, and those that reduce increased skeletal muscle tension) had the highest Hg content (Me = 4.4 µg/kg). Antibiotics (Me = 2.2 µg/kg), antidiarrhoeals (Me = 1.4 µg/kg), and analgesics/antipyretics/anti-inflammatories (Me = 1.1 µg/kg) followed in descending order. For medicines that are protective against heart and blood vessel diseases (Me = 0.2 µg/kg), as well as medicines from the antibacterial, antiviral, antifungal, bactericides group (Me = 2.3 µg/kg), the Hg levels were found to be the lowest in our study. However, Kowalski and Frankowski [37] reported results that were about 10 times higher than ours.

Puścion-Jakubik et al. [38] obtained Hg contents in DS collected in Poland that were very similar to ours, with a mean concentration of 3.37 μg/kg and a median of 1.69 µg/kg. Our study found values of 2.7 µg/kg and 2.0 µg/kg for DS, respectively. Puścion-Jakubik et al. [38] found that preparations responsible for glucose control (used for lowering glucose levels) had the highest median value of 5.94 µgHg/kg among the different types of DS studied, while preparations with a detoxifying effect (detoxifying supplements) had the lowest Hg levels (Me = 0.64 µg/kg). In our study, probiotics and minerals had the lowest levels of Hg among DS, with 0.8 μg/kg and 0.9 μg/kg, respectively.

Another study on DS available in the Polish market was conducted by Socha et al. [39]. Like our study, none of the tested preparations exceeded acceptable standards. The range of variation was 0.10–47.99 µg/kg [39], which was greater than what we obtained (0.07–18.36 µg/kg), but similar to the range of variation for medicines (0.12–57.42 µg/kg). According to Socha et al. [39], the DS with the highest Hg content was found to affect the urinary tract (9.98 µg/kg), while the lowest was found in stimulant preparations (2.37 µg/kg). In our study, the highest Hg contents in the DS groups were 3.9 µg/kg slimming preparations and for hair, nails, and skin improvement preparations.

The scientific literature contains a significant amount of information regarding the presence of Hg in medicinal plants [40–43] and plant raw materials used in DS [44, 45]. Hg can hinder photosynthetic activity, slow down growth processes, and impede root development in plants [41]. In a study of 14 medicinal plant species from certified producers in Romania, approximately 28% of samples exceeded the WHO limit of 0.2 mg/kg for Hg [41]. The plants with the highest concentrations of Hg were wormwood (*Artemisia absinthium* L.), dandelion (*Taraxacum officinale* Webb.), and mullein (*Verbascum thapsus* L.), ranging from 650 to 692 µg/kg [41]. Toxic elements present in dried plants can enter into the human body upon ingestion. Therefore, it is necessary to quantify toxic elements for quality control of phytotherapeutic products. Studies of herbal DS from Nigeria have also reported exceeding the limit for Hg content [46].

In contrast, studies on herbs with food and medicinal uses, such as chia (*Nigella sativa*), cumin (*Cuminum cyminum*), Ceylon cinnamon tree (*Cinnamomum zeylanicum*), conducted in Pakistan, indicate that the concentrations of Hg ranged from 2.25 to 3.75 µg/g and the calculated EDI (Estimated Daily Intake) value was below the recommended daily intake limit [43]. Similarly, studies by Singh et al. [40], Sindhu and Beena [47] from India, and Begum et al. [42] from Pakistan found that the presence of Hg in medicinal plants was below permissible levels.

A Polish study [44] of DS containing terrestrial plants or microalgae found Hg in 29.3% of samples. The average Hg content in samples with a plant component was 5 µgHg/kg and with microalgae was 3 µg/kg. In this study, the average Hg content in DS with the plant component was 3.9 µg/kg. This group of DS had the highest levels of Hg of all DS studied. Smaller amounts of Hg were found in vitamin-mineral DS (1.8 μg/kg) and others (1.1 μg/kg). Kowalski and Frankowski’s study [37] did not differentiate the plant-based DS group, and the vitamin supplement group had the highest Hg content.

Dolan et al. [48] reported a wide range of Hg concentrations in DS from the USA, mainly those containing the plant component, ranging from 80 to 16,800 µg/kg. The median value for these samples was less than 80 µg/kg, and Hg exposure was determined to be below the tolerable intake of 5 µg/kg body weight/week [48]. In the herbal DS we studied, the Hg content was much lower, ranging from 0.1 to 18.4 µg/kg.

Other studies conducted in the USA have shown that DS containing ginkgo biloba, fish oil, and echinacea have higher levels of Hg, while St. John’s wort products have lower levels of Hg. The fish oil product had the highest level of Hg at 123 µg/kg [49]. The studies on oils products did not confirm this relationship. It was found that oils of vegetable origin had the highest Hg content at 0.218 μg/kg, followed by cod liver oil at 0.106 μg/kg and shark liver oil at 0.065 μg/kg [50]. The study suggests that vegetable-based oil products have a higher Hg content, which may be due to exposure from the soil and other sources [50].

Tumir et al. [51] conducted a study on the most common DS of various origins (vitamin, mineral, vegetable, and animal) available in Croatia. The study found that the range of Hg content was 0.02–0.12 µg/g, Me = 0.05 µg/g. In comparison, our study found a smaller range of Hg at 0.1–56.4 µg/kg, with the same median of 1.2 µg/kg.

Korfali et al. [52] conducted a study on the Hg content of DS available in Lebanon. They found that this element was present in all preparations, with an average of 80 µg/kg and a range of variation of 10–550 µg/kg. The DS tested contained various ingredients, including vitamins, minerals, and raw plant material, and came from different countries, mainly the USA. The levels of Hg detected by Korfali et al. [52] were significantly higher than those found in our study.

Torović et al. [53] conducted a study on herbal DS in Serbia and found that 38.5% of the samples contained Hg, with the highest concentration being 70.5 µg/kg. Statistically significant differences were observed between the paediatric and adult DS groups, with higher amounts of Hg found in the adult samples (*p* = 0.017). The intake of Hg in the analysed DS did not exceed acceptable standards. When assessing health risks, it is crucial to take into account the potential impact of daily dietary intake of Hg. Our study did not provide a breakdown of the DS analysed by age group, but it did reveal a maximum Hg concentration of 18.4 µg/kg.

After analysing the available literature, it is evident that the Hg content of medicines and DS varies significantly [37–39, 43–46, 48–56]. Different groups of drugs and DS have been shown to have minimum and maximum values for Hg. In most studies, the amounts of Hg in finished preparations and medicinal plants have been found to be within acceptable standards. However, there are cases where this is not the case. Prolonged use of preparations increases the risk of Hg exposure. Due to the widespread public confidence in the efficacy and safety of natural/herbal products, it is important to suggest and develop stringent controls based on legal standards. This is particularly important given the aggressive promotional marketing, the trend towards improved health and beauty, the simultaneous use of multiple supplements, possible interactions, and the large spectrum of potential contaminants.

Due to the continuous and significant increase in the number of DS registered in Poland, the European Union (EU) [8], and worldwide [48, 49], there is a need for an increase in the number of quality studies on these products. Consumer awareness campaigns on DS, possible drug interactions, and adverse effects should also be introduced. The wide variety of DS available on the market and the rapid changes in their availability make it impossible to compare the occurrence of Hg over a longer period of time.

## Conclusions

The Hg content was higher in medicines, then in DS (3.9 vs. 2.7 µg/kg), the differences were no statistically significant.

None of the tested preparations (medicine and DS) exceeded acceptable standards.

DS of herbal origin had a significantly higher Hg content compared to vitamin-mineral supplements and others. Among the different intended uses of DS, slimming and skin, hair, and nail support preparations had the highest Hg concentrations, while minerals had the lowest.

Of all the medicines tested, prescription contained a higher amount of Hg than OTC.

It is advisable to exercise caution in the choice of medicines and DS due to possible contaminants and interactions, and to use them for a limited period of time only.

## Data Availability

No datasets were generated or analysed during the current study.
